# Self-Care Ability of Patients With Severe Mental Disorders: Based on Community Patients Investigation in Beijing, China

**DOI:** 10.3389/fpubh.2022.847098

**Published:** 2022-06-01

**Authors:** Chen Chen, Yun Chen, Qingzhi Huang, Shengming Yan, Junli Zhu

**Affiliations:** ^1^School of Public Health, Capital Medical University, Beijing, China; ^2^Research Center for Capital Health Management and Policy, Beijing, China; ^3^The National Clinical Research Center for Mental Disorders and Beijing Key Laboratory of Mental Disorders, Beijing Anding Hospital, Capital Medical University, Beijing, China; ^4^Advanced Innovation Center for Human Brain Protection, Capital Medical University, Beijing, China; ^5^Beijing Institute of Mental Health, Beijing, China; ^6^Department of Sociology, Peking University, Beijing, China

**Keywords:** severe mental disorders, self-care ability, influence factors, daily basic activity, housework, social function

## Abstract

**Background:**

Severe Mental Disorders have become a topic of increasing interest in research due to their serious consequences for the quality of life and functioning. In the pages that follow, it will be argued that the self-care ability and its influencing factors among patients with severe mental disorders in Beijing, according to the questionnaire survey in 2019.

**Methods:**

Proportionate stratified sampling was used to select representative patients as samples. The demographic characteristics of were obtained from the Management Information System for Severe Mental Disorders and the questionnaires. The self-care ability was measured by self-made scales. Descriptive statistics, *t*-test, and multiple linear regression were used to analyze the data.

**Results:**

We surveyed 662 people and found that the deficiency of self-care ability is common in patients with severe mental disorders. Self-care ability was positively correlated with educated levels and guardian takes care of alone, and negatively correlated with age, course of disease and physical disease (*P* < 0.05). From a dimensional perspective, the daily basic activity was positively correlated with educated levels and negatively correlated with physical disease (*P* < 0.05); the housework ability was positively correlated with gender, educated levels and medication adherence, and negatively correlated with source of income and physical disease (*P* < 0.05); the social function was positively correlated with educated levels, guardian takes care of alone and medication adherence, and negatively correlated with age, source of income, course of disease and physical disease (*P* < 0.05).

**Conclusion:**

The self-care ability of patients with severe mental disorders is affected by many factors, including patient characteristics and social factors. Therefore, targeted interventions are needed to help patients restore their self-care ability, which requires the joint efforts of the government and the whole society.

## Introduction

The World Health Organization defines Severe Mental Disorder (SMD) as a group of conditions that include moderate to severe depression, bipolar disorder, and schizophrenia and other psychotic disorders (1). The patients usually have moderate to severe impairment of work or non-work activities, as well as impairments in social function and daily basic mobility (2). To make matters worse, SMD is usually accompanied by physical diseases, such as cancer, cardiovascular disease, diabetes, stroke, tuberculosis, and AIDS, etc. (3). The WHO claims that around 1 in 9 people in settings affected by conflict have a moderate or severe mental disorder, and SMD patients die 10 to 20 years earlier than the general population (4). In addition, depression disorders are listed as one of the top 10 causes of DALY (5). Because of the disease, patients often have obstacles in personal wellbeing, social relationships, and work productivity (6). WHO said the depression and anxiety cost the global economy US$ 1 trillion each year (7). Mental, neurological, and substance use disorders also make up 10% of the global burden of disease and 30% of the non-fatal disease burden (4). In China, there are six types of SMD: schizophrenia, bipolar disorder, paranoid disorder, schizoaffective disorder, mental disorders in epilepsy and mental retardation (8). Studies have mentioned that between 2012 and 2030, the loss of productivity due to mental illness in China will reach US$900 million (9). So, improving the self-care capacity of people with mental disorders is a necessary measure to promote population health and reduce the burden on patients, families, and society.

To better help people with mental disorders recover, WHO launched *the Comprehensive Mental Health Action plan 2013–2020* in 2013 (10). This plan supports the establishment of organizations to help people with mental disorders and psychosocial disabilities. It also suggests that a multi-sectoral collaboration is required to offer assistance at different stages of the life course such as educational opportunities, employment, participation in community activities, etc. In China, the national “686 Program” (also called “*Central Government Support for the Local Management and Treatment of Serious Mental Illness Project”*) provided treatment and assistance for patients with poor families and implemented “unlocking” actions for patients locked at home, to increase the rate of patients' recovery and return to society (11). In response to the call of promoting the self-care ability of SMD patients to recover, a series of policies focusing on community rehabilitation have been issued all over the country. Jilin, Jiangsu, Jiangxi and other places provide rehabilitation services such as labor skill and social ability training for patients through community pilots. Such a community-based model of mental disorders to help patients recover also has been implemented in the United States, Britain, France, Japan, and other countries (12, 13). Hunan Province, drawing on the experience of the United States (14), introduced more humane care called “the clubhouse model.” This model regards patients as members and helps patients actively integrate into society by establishing an open, relaxed and positive rehabilitation environment.

Beijing, as the capital of China, also attaches great importance to the rehabilitation of people with mental disorders. According to *the 2020 Annual Report on the Monitoring of Severe Mental Illness*, the number of SMD patients registered in Beijing is 81,347 (15), and the cumulative number of patients has been increasing in recent years. *The Implementation Program for precision care for persons with disabilities in Beijing (2018-2020)* proposes a “1+6” plan system, which requires communities to have the infrastructure to provide rehabilitation training, self-care training, and social adaptability counseling. In 2018, Beijing launched a pilot construction of a psychosocial service system to help patients recover their physical and mental health more comprehensively with the help of the community. Because of the financial burden and care burden caused by severe mental disorders, many measures have been implemented. If we can improve the self-care ability of patients, it is not only a good thing for patients themselves or their families, but also of great significance to maintain social stability. However, only a few publications are researching the self-care ability of SMD patients in China. Li et al. (16) investigated the self-care ability of patients with mental disorders in three medical institutions, and the results showed that patients in the hosting centers and rehabilitation centers had strong self-care ability. Chen et al. (17) investigated the self-care ability and social support of elderly patients in the community, and the results showed that the elderly have impaired daily life function and lack social support. In the past ten years, there has been less research on the self-care ability of SMD patients abroad. Based on the necessity for further research, this study aims to investigate the current situation of self-care ability in SMD people and the factors that affect it, in the hope of obtaining more targeted assistance to patients and providing the basis for improving relevant policies.

## Materials and Methods

### Sample and Data

In Beijing, when the SMD patients were diagnosed by psychiatric hospitals, their information was recorded in the Beijing Municipal Management Information System for Patients with Severe Mental Disorders in order to provide assistance. We used this system to select a representative sample to conduct a cross-sectional study of SMD patients in Beijing. The demographic characteristics and self-care ability of patients were obtained from the System and questionnaires (The questionnaire can be found in Multimedia Appendix 1 and the raw data can be found in Appendix 2). To ensure the quality of the investigation, we recruited medical students with social medical background as investigators. Investigators are trained for 1 day on the application of the scale and data collection before the commencement of the investigation. The investigators are required to explain the purpose of this study to ensure that all respondents participated voluntarily and signed informed consent. This study passed the ethical review conducted by the Medical Ethics Committee of Capital Medical University. Questionnaires are distributed one-on-one and answered anonymously, guardians are interviewed face-to-face, and questionnaires are unified and collected by investigators.

**Figure 1:**
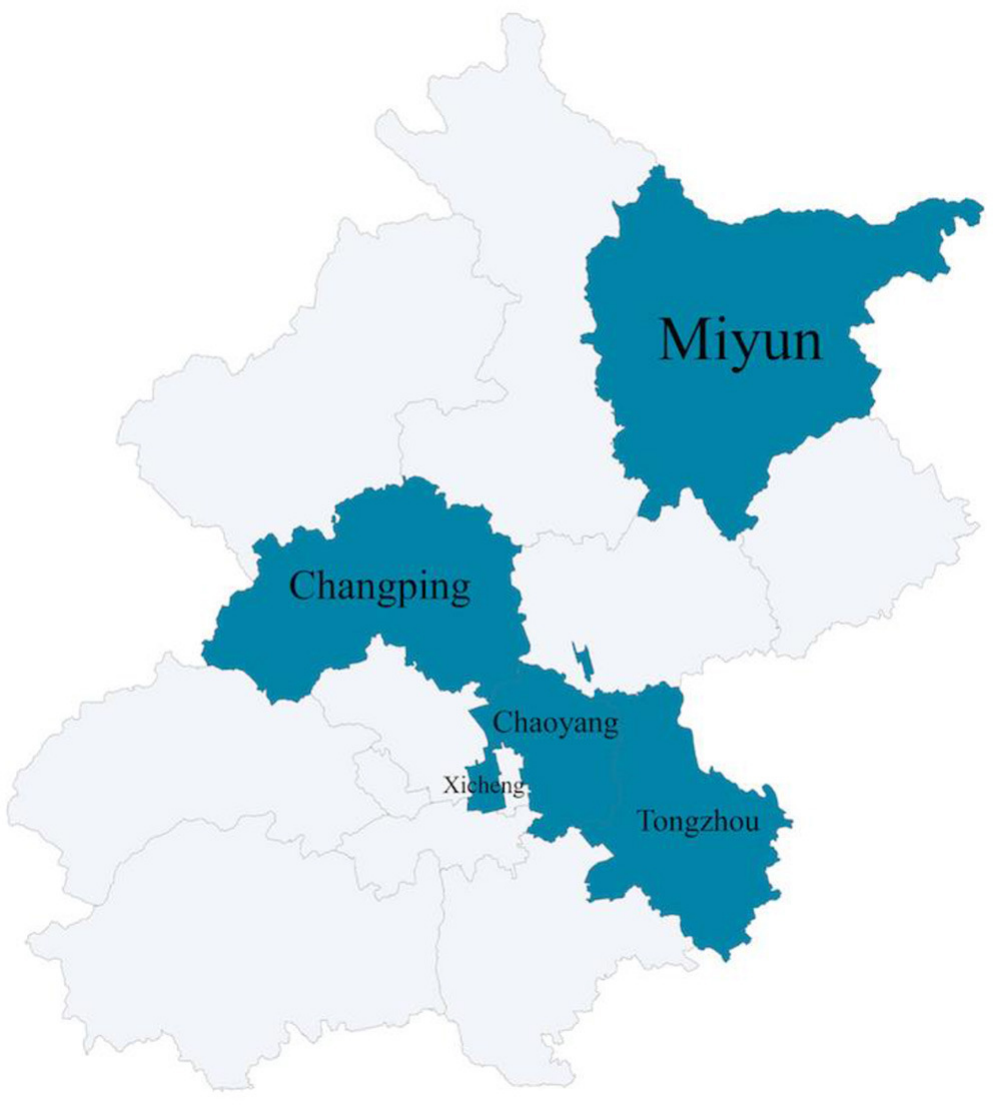
Districts sampling situation.

The samples were obtained by proportionate stratified sampling in SMD Patient Management System using the following steps: first, the total sample size and sampling districts was determined. Four districts were selected according to the functional areas of Beijing (including the core area of the capital function, Urban Fringe's Sustainable Development, urban development zone, ecological conservation area), plus Tongzhou District (deputy city center). As a result, XiCheng, ChaoYang, ChangPing, TongZhou, and MiYun were selected. The selected districts are shown in Figure 1. Second, the sample size of the district was calculated based on the proportion of patients in each district, and the sample districts are divided according to urban and rural areas, with a random selection of 1 street and 1 township from each of the two areas. Third, the sample size of the street/township was determined based on the ratio of the number of people in each street/township, and then to make the sample representative, patients were grouped by gender-age and the number of patients in each group was calculated based on the gender-age ratio in Beijing in 2017. Finally, the patients were selected randomly according to the calculated number of samples of each group. Because the guardian is the main caregiver of the patient, who has a better understanding of the patient's self-care ability and more accurate evaluation, so the questionnaire was answered by the guardian.

The inclusion criteria for survey subjects of this study are as follows: (1) Patients have been registered in the management system and diagnosed with one of the six types of severe mental disorders (the six severe mental disorders are schizophrenia, bipolar disorder, paranoid disorder, schizoaffective disorder, mental disorders in epilepsy and mental retardation); (2) The guardian is the primary caregiver of the patient and has a sound cognitive function; (3) The patient and guardian are volunteered to participate in the survey. The following exclusion criteria were applied: Patients who have not been profiled in the management system for SMD patients. Nine hundred and thirty questionnaires were distributed in five districts of Beijing, of which 910 responded, accounting for 97.9%. After deleting the missing values and illogical responses, the final sample consisted of 662 people.

### Instruments

In this research, the self-designed questionnaire was used to evaluate the self-care ability of patients. There are several steps to select the items for the questionnaire. First of all, the questionnaire was designed by referring to the Activities of Daily Living (ADL) scale, which has been used in many previous studies and proved to have good reliability and validity (18, 19), and the factors that might affect the self-care ability. Secondly, the questionnaire had been revised after experts' evaluation. Thirdly, a pre-survey was executed with a small sample to further modify the instruments. The final questionnaire is divided into two parts. The first part is the demographic characteristics. Previous studies have shown that factors such as age, course of illness, and gender have an impact on mental health (20, 21). Taş (22) showed that self-care ability point average of individuals who were single, did not have children, or had a member of family with mental disease was significantly lower. Based on experience, information on the patient's gender, age, household registration, education level, etc. was collected from System and questionnaires. The data on medication adherence and mental stability need to be rated by the guardian on a Likert scale, as well as fill in information about the patient's diagnosed physical disease through a questionnaire. The second part is the evaluation of self-care ability by referring to the ADL scale. The scale consists of 18 items and is subdivided into 3 dimensions. These three dimensions are daily basic activities (9 items, including meals, dressing, bathing, etc.), household activities (3 items, including sweeping, cooking, and laundry), and social activities (6 items, including shopping, making phone calls, managing finances, etc.) (23, 24). Each item is rated as “patients can't do it at all,” “patients need help from others to do it,” “patients themselves can do it completely,” assigned 1 to 3, the resulting score ranges between 18 and 54, the higher the score, the better self-care ability.

Cronbach's α was used to measure the reliability and Kaiser–Meyer–Olkin (KMO) was used to measure the validity. The results have shown that Cronbach's α is 0.940 and the KMO was 0.929, which demonstrated high levels of reliability and construct validity.

In order to make the mean values comparable across dimensions, the scores are standardized with 100 as the full score. Descriptive statistics were used for reporting the characteristics of the sample, as frequencies and percentages for count data, mean values, and standard deviations (SD) for measurement data. To examine the impact of demographic characteristics and other factors on the self-care ability of patients, there are two steps in statistical analysis. The first step is to use the *t*-test to compare self-care ability according to two-state independent variables (such as male and female) and ANOVA was used to compare self-care ability according to more than two states variables (such as educated levels). The second step is multiple linear regression. Since the results of the regression analysis did not require comparison across dimensions, the unstandardized scores were used for the analysis to improve accuracy. Self-care ability is divided into three dimensions: daily basic activities, household activities, and social activities. So, multiple linear regression was used in each dimension. All statistical analysis is achieved through SPSS 26.0 and variables are considered statistically significant at the typical 95 % level.

## Results

### Description of the Basic Characteristics of the Sample

The basic characteristics of all respondents in this study are shown in Table 1. The sample consisted of 348 women and 314 men, ranging in age between 16 and 97 years. Most of them were over 40 years of age, accounting for 79.2%. In addition, 82.3% of the patients were urban residents, 16.5% of them had a college degree or above, 55.9% of them had a disease course of 20 years or longer. There were more patients with schizophrenia and bipolar disorder, of which 63.4% were schizophrenic, 12.4% were with bipolar disorder, 47.7% had the physical disease, and 69.3% of them used pensions or dole as the main source of income.

**Table 1:** Description of respondent characteristics.

**Variable**		***n***	**%**
Gender	Male	314	47.4
	Female	348	52.6
Age (years)	<40	138	20.8
	40 60	278	42.0
	≥60	246	37.2
Household register	Urban residents	545	82.3
	Rural residents	117	17.7
Education	Primary or less	182	27.5
	Junior	195	29.5
	High	176	26.6
	College degree and above	109	16.5
Source of income	Wage	170	25.7
	Pension	374	56.5
	Dole	85	12.8
	Other sources	33	5.0
Guardian takes care of alone	Yes	341	51.5
	No	321	48.5
Course of disease (years)	<20	292	44.1
	20 40	253	38.2
	≥40	117	17.7
Diagnosis	Schizophrenic	420	63.4
	Bipolar disorder	82	12.4
	Other mental disorders	160	24.2
Physical disease	Yes	346	47.7
	No	316	52.3
Mental stability	Low	123	18.6
	General	161	24.3
	High	378	57.1
Medication adherence	Low	131	19.8
	General	248	37.5
	High	283	42.7
Total		662	100

### Variables Assignments and Univariate Analysis Results

The variable assignment situation and the univariate analysis affecting self-care ability are shown in Table 2. It can be seen that the self-care ability of patients had different degrees of damage. Among them, the scores of basic daily activities were high, while the scores of domestic activities and social functions were low, indicating that these two abilities were seriously damaged. The results also indicated that: age, education, course of disease, and physical disease were statistically significant in all dimensions. Mental stability, which means the mood fluctuation is controllable and will not affect the daily life, was statistically significant only in total ability, gender was statistically significant only in the dimension of housework activities, and medication adherence was statistically significant only in the dimension of social function. In terms of disease type, compared to other diseases, patients with schizophrenia and bipolar disorder have better overall self-care ability and better ability to perform basic daily activities than patients with other mental illnesses; bipolar patients have better social functioning than patients with other mental illnesses.

**Table 2:** Variables assignments and univariate analysis results.

**Variable**		**Assignment**	**Overall ability**		**Each dimension**					
					**Daily basic activity**		**Housework**		**Social function**	
			**Mean ±SD**	**t/F**	**Mean ±SD**	**t/F**	**Mean ±SD**	**t/F**	**Mean ±SD**	**t/F**
Gender	Male	0	81.12 ± 14.50	−1.421	92.74 ± 12.41	0.734	68.71 ± 27.64	−3.820^**^	69.88 ± 19.24	−1.465
	Female	1	82.83 ± 16.37		91.96 ± 14.95		76.75 ± 26.46		72.19 ± 21.02	
Age (years)	<40	Assign a value of 1–3 from short to long	83.99 ± 14.25	18.394^**^	93.55 ± 11.89	11.703^**^	73.10 ± 26.87	7.840^**^	75.08 ± 18.88	20.779^**^
	40 60		85.12 ± 13.47		94.63 ± 10.86		77.29 ± 26.16		74.78 ± 18.77	
	≥60		77.41 ± 17.24		89.05 ± 16.84		67.93 ± 28.06		64.70 ± 20.95	
Household register	Urban residents	0	82.81 ± 15.46	2.837^**^	92.64 ± 13.68	1.227	73.61 ± 27.20	1.373	72.67 ± 19.98	4.380^**^
	Rural residents	1	78.34 ± 15.36		90.91 ± 14.29		69.80 ± 27.67		63.77 ± 19.71	
Education	Primary or less	Assign a value of 1–4 from low to high	75.18 ± 16.15	22.568^**^	88.15 ± 15.76	10.209^**^	64.04 ± 27.84	11.288^**^	61.29 ± 20.24	29.032^**^
	Junior		81.54 ± 16.06		92.00 ± 14.96		73.16 ± 27.11		70.05 ± 20.16	
	High		86.98 ± 12.66		95.39 ± 10.58		79.92 ± 25.02		77.9 ± 17.47	
	College degree and above		86.28 ± 13.31		94.97 ± 10.68		76.14 ± 26.57		78.33 ± 17.20	
Source of income	Wage	Set 3 dummy variables	84.92 ± 14.48	5.070^**^	93.94 ± 11.60	1.495	78.82 ± 24.80	5.024^**^	74.44 ± 20.00	6.472^**^
	Pension		82.05 ± 15.59		92.18 ± 13.99		72.25 ± 27.75		71.74 ± 20.09	
	Dole		77.45 ± 15.82		90.58 ± 16.36		65.62 ± 27.46		63.66 ± 19.31	
	Other sources		78.56 ± 16.45		90.23 ± 14.60		69.36 ± 29.13		65.65 ± 20.00	
Guardian takes care of alone	No	0	80.46 ± 16.04	−2.514*	91.30 ± 15.07	−1.864	71.37 ± 27.58	−1.436	68.76 ± 20.45	−2.902^**^
	Yes	1	83.49 ± 14.90		93.30 ± 12.42		74.42 ± 27.00		73.29 ± 19.75	
Course of disease (years)	<20	Assign a value of 1-3 from short to long	84.96 ± 14.16	12.758^**^	94.07 ± 12.18	6.498^**^	77.39 ± 25.32	7.354^**^	75.09 ± 18.75	15.082^**^
	20 40		81.02 ± 15.40		91.99 ± 13.67		70.13 ± 28.24		70.02 ± 20.20	
	≥40		76.82 ± 17.45		88.73 ± 16.90		67.90 ± 28.54		63.43 ± 21.37	
Diagnosis	Schizophrenic	Set 2 dummy variables	82.69 ± 15.50	6.943^**^	92.55 ± 13.93	4.044*	74.04 ± 26.77	3.141	72.22 ± 19.63	8.298^**^
	Bipolar disorder		85.59 ± 12.87		95.39 ± 8.83		76.15 ± 26.65		75.60 ± 19.86	
	Other mental disorders		78.43 ± 16.25		90.18 ± 15.20		68.40 ± 28.62		65.83 ± 20.96	
Physical disease	No	0	84.77 ± 14.44	4.821^**^	94.01 ± 12.35	3.274^**^	76.87 ± 25.93	3.907^**^	74.85 ± 18.78	5.074^**^
	Yes	1	79.01 ± 16.13		90.49 ± 15.03		68.63 ± 28.15		66.98 ± 20.93	
Mental stability	Low	Assign a value of 1–3 from low to high	81.66 ± 15.35	3.255^*^	91.97 ± 13.95	0.967	74.55 ± 26.63	1.512	69.76 ± 19.57	6.173
	General		80.29 ± 16.01		91.56 ± 14.87		70.56 ± 27.87		68.25 ± 20.40	
	High		83.70 ± 15.05		93.18 ± 12.71		74.28 ± 27.06		74.20 ± 19.97	
Medication adherence	Low	Assign a value of 1–3 from low to high	80.53 ± 15.72	1.728	92.11 ± 14.23	0.189	70.18 ± 28.03	0.982	68.33 ± 20.16	4.015^*^
	General		80.89 ± 15.72		91.85 ± 14.53		72.39 ± 27.30		68.70 ± 20.10	
	High		82.99 ± 15.35		92.61 ± 13.36		74.07 ± 27.07		73.01 ± 20.11	
Total		662	82.02 ± 15.53		92.33 ± 13.80		72.94 ± 27.30		71.09 ± 20.21	

* *0.05*,

** *0.01.*

*The bold values is that the variables are statistically significant; 0,1 is the assignment of variables*.

### Multiple Linear Regression Analysis Results

The multiple linear regression was used to analyze the impact of demographic characteristics, disease-related characteristics, and medication adherence on self-care ability. The results are shown in Table 3. Patients who were young, highly educated, had a short course of illness, no physical illness and a guardian caring for them alone had a high level of comprehensive self-care; Patients with a high level of education and no physical diseases had a greater ability to perform basic daily activities; Patients who were female, had a high level of education, relied on wage as a source of income, had no physical diseases and had good adherence with medication were more able to perform household activities; Patients who were younger, had a high level of education, relied on wage as a source of income, had a short course of illness and good adherence with medication, did not have a physical disease and were cared for alone by a guardian had greater social functioning. In general, educational level and physical disease had an impact on all dimensions of self-care. Regression coefficients of each dimension passed the test at the 0.05 significance level.

**Table 3:** Multiple linear regression model results.

**Variables**	**Overall ability**		**Different dimensions**					
			**Daily basic activity**		**Housework**		**Social function**	
	**B**	**OR (95%CI)**	**B**	**OR (95%CI)**	**B**	**OR (95%CI)**	**B**	**OR (95%CI)**
Gender	1.005	2.73 (0.81–9.21)	−0.226	0.79 (0.45–1.40)	0.728^**^	2.07 (1.43–2.98)	0.504	1.65 (0.99–2.76)
Age	−1.042^*^	0.35 (0.14–0.88)	−0.418	0.65 (0.42–1.01)	−0.040	0.96 (0.72–1.26)	−0.584^**^	0.55 (0.37–0.82)
Household register	0.224	1.25 (0.21–7.35)	0.381	1.46 (0.64–3.33)	0.140	1.15 (0.67–1.96)	−0.297	0.74 (0.35–1.56)
Education	1.628^**^	5.09 (2.61–9.91)	0.509^**^	1.66 (1.22–2.26)	0.349^**^	1.41 (1.15–1.73)	0.770^**^	2.15 (1.63–2.86)
**Source of income**								
Pension	−0.567	0.56 (0.12–2.52)	−0.141	0.86 (0.43–1.73)	−0.380	0.68 (0.43–1.07)	−0.046	0.95 (0.50–1.79)
Dole	−2.019	0.13 (0.01–1.10)	−0.343	0.70 (0.26–1.90)	−0.787^*^	0.45 (0.24–0.86)	−0.889	0.41 (0.16–1.00)
Other sources	−2.974	0.05 (0–0.98)	−0.926	0.39 (0.10–1.56)	−0.799	0.44 (0.18–1.09)	−1.250^*^	0.28 (0.08–0.99)
Guardians takes care of alone	1.270^*^	3.56 (1.05–12.06)	0.454	1.57 (0.89–2.77)	0.188	1.20 (0.83–1.74)	0.628^*^	1.87 (1.12–3.13)
Course of disease	−0.972^*^	0.37 (0.15–0.92)	−0.273	0.76 (0.50–1.15)	−0.259	0.77 (0.58–1.01)	−0.441^*^	0.64 (0.44–0.93)
**Diagnosis**								
Bipolar disorder	0.166	1.18 (0.17–7.94)	0.390	1.47 (0.60–3.57)	−0.241	0.78 (0.44–1.39)	0.017	1.01 (0.45–2.27)
Other mental disorders	−0.731	0.48 (0.10–2.26)	−0.291	0.74 (0.36–1.53)	−0.101	0.90 (0.56–1.44)	−0.339	0.71 (0.37–1.36)
Physical disease	−2.297^**^	0.10 (0.02–0.34)	−0.656^*^	0.51 (0.29–0.92)	−0.642^**^	0.52 (0.36–0.76)	−0.999^**^	0.36 (0.21–0.62)
Mental stability	0.156	1.16 (0.46–2.91)	0.114	1.12 (0.73–1.71)	−0.121	0.88 (0.67–1.16)	0.163	1.17 (0.80–1.72)
Medication adherence	0.803	2.23 (0.92–5.39)	0.059	1.06 (0.70–1.59)	0.274^*^	1.31 (1.00–1.71)	0.470^*^	1.59 (1.10–2.32)
Constant	42.952		25.055		6.120		11.777	
Adj *R^2^*	0.132^**^		0.053^**^		0.083^**^		0.180^**^	

* *0.05*,

** *0.01*.

*The bold values is that the variables are statistically significant*.

## Discussion

The status of people with severe mental disorders in many countries is not very good. Berlim et al. (25) suggested that the quality of life in patients with mental disease is poor in Brazil, which is true in the United States, Germany, and South Africa (26–28). This study also found that the self-care ability of patients with severe mental disorders in Beijing was impaired. The results showed that most patients were older, had long illnesses, and nearly half had the physical disease at the same time. The characteristics of respondents are similar to Fleury et al. (29) and Shumye et al. (30). The average scores of basic daily activities are more than 90, indicating that patients have a strong ability to eat, dress, and other simple behaviors generally. The weakening of housework and social function is more serious, with an average score of <80, which is much lower than the ability of basic daily activities. Although this study did not explore the reasons for the weakening of ability, it has been analyzed in previous literature. Some scholars suggested that one of the reasons is the lack of perseverance and enthusiasm for life, resulting in the inability or unwillingness to participate in housework and social activities actively (31, 32). On the other hand, prejudice and discrimination are common in society, and the patients have a sense of stigma (33) makes them lack social identity (34), resulting in more serious damage to social function. So, it is necessary to popularize mental health knowledge in the whole society. In this way to enhance the awareness of mental illness prevention and change people's prejudice against mental disorders. This research also found that patients with schizophrenia and bipolar disorder have better self-care ability compared to other psychiatric disorders, and Lan et al. (35) also indicated that patients with these two disorders have better treatment compliance, which suggests that we can improve patients' self-care ability by increasing compliance. Therefore, mental health knowledge should be further disseminated to make patients subjectively willing to accept treatment, while objectively promoting patients to accept treatment through follow-up visits by community physicians.

This study found that some demographic characteristics have a significant impact on patients' self-care ability. First of all, the patient's educational level affects all the dimensions of self-care abilities including daily basic activity, housework, and social function, which showed the higher the level of education, the stronger the self-care ability. Caron (36) has pointed that patients with a high level of education have a higher awareness of the disease and are more willing to accept treatment and cooperate with communities in rehabilitation activities (36). Other factors also affect the patient's self-care ability but have different effects on the different dimensions. For example, gender only affects housework ability, while age only affects social ability. Women are often the main contributors to domestic activities in traditional families in China, so female patients have greater housework ability (36). The older patients have poorer memory and understanding ability, which reduces their willingness and ability to participate in social activities. This inference was similar to a study by (20) who found that age was a factor influencing the quality of life among older people in Songkhla (20). Therefore, classified management can be implemented for patients. For patients with higher education, the goal is to promote their self-care ability to return to the normal level. Because the patients with higher education have higher treatment compliance than other patients, who are easier to restore health. For the elderly vulnerable group, the goal is to help them improve their self-care ability as much as possible. The community should visit the patients regularly to understand their physical and mental status and medication. Welfare institutions such as nursing homes should pay more attention to the mental health of the elderly because the elderly are more likely to have psychological problems due to the lack of family companionship.

Another finding of this study is that the longer the course of the disease, the weaker the patient's ability to do housework and social function. Mental disorders, as a chronic disease, have the characteristics of long course and frequent recurrence (37). Patients tend to lose confidence and become negative, gradually changing from complementary treatment to resistance therapy in the process of long-term treatment. In addition, previous studies have shown that mental disorders are often associated with physical diseases (38–40). Impaired physical and mental health prevents patients from engaging in housework and complex social activities (41). So, clinical research needs to pay attention to complications and promote the recovery of overall function through combined therapies. What's more, it is necessary to promote the reorganization of health care to facilitate treatment and recovery of people who are struck by comorbid mental and physical disorders.

Insist of drug treatment can help improve the symptom. Ansari et al. (42) study has found that improving medication compliance is of great significance to the management of schizophrenia. But researches have shown that the medication compliance of patients with mental disorders is generally poor (43–46). Thus, interventions to improve medication adherence among SMD patients are urgently needed. Sun et al. (47) pointed out that there was a significant correlation between economic and medication compliance. So, the Community Free-Medication Service policy (CFMS) was implemented in Beijing in 2013 to reduce the financial burden caused by taking medicine. The existing research also suggested that family and community play an important role in improving medication compliance (48, 49). Therefore, health education should be carried out for patients and families. At the same time, we should also improve the quality of doctors and community service. Through case management and medication consultation to improve patient's medication compliance.

The main contributions of this study are as follows: firstly, the sample was selected in the community rather than in psychiatric hospitals like most previous clinical studies. For the reason that with the development of deinstitutionalization, SMD patients gradually return to the community and the focus should be on the community. Secondly, the study described the overall situation of mental disorders and the participants covered all kinds of SMD people, rather than patients with a certain type of mental disorders (such as bipolar disorder, depression, etc.), the results are more comprehensive. In addition, compared with developed countries such as the United States, Britain, and Germany in previous studies (50–52), this article provides empirical evidence under different research context. China is a country with low and medium economic development levels with a large population. Unlike other developing countries such as India (53), China's political system has its particularity, and so does its medical and health care system. This study also has some limitations. One is that the questionnaire was answered by guardians for the reason that they know more about the patient's self-care ability with the perennial care. The view of evaluation will be more comprehensive if the doctor's opinions are added. Besides, due to the limited research time and resources, this study is a simple cross-sectional design. It is difficult to explain the causal relationship between mental disorders and impaired self-care ability. What's more, mental disorders often have complications, which can also damage the patient's self-care ability. What kind of disease impairs the self-care ability needs more in-depth research. At the same time, patients with serious impairment of self-care ability may be inconvenient to be investigated, so there is an error between the survey results and the actual situation. After that, the people selected in this survey can be followed up regularly to further explore the relationship between mental disorders and self-care ability.

## Conclusion

A cross-sectional study was used to explore the self-care abilities of SMD patients. The results showed that most patients had impaired self-care ability and it is influenced by many factors. It is suggested to strengthen the assistance to vulnerable groups, and pay attention to the psychological intervention of patients. The impairment of self-care ability caused by complications also needs attention. There should be a more meticulous comprehensive treatment plan. Family and community intervention can be used to improve patients' medication compliance and help patients recover their self-care ability.

## Data Availability Statement

The original contributions presented in the study are included in the article/Supplementary Materials, further inquiries can be directed to the corresponding author.

## Ethics Statement

The protocol of this study was approved by the Medical Ethics Committee of Capital Medical University (No: Z2020SY123). All respondents were voluntary and written informed consent was obtained. All data collection is anonymous.

## Author Contributions

JZ contributed to the conception and design of the study. JZ, CC, YC, QH, and SY organized the data collection. CC performed the statistical analysis. JZ, YC, and CC wrote sections of the manuscript. All authors contributed to the manuscript revision, read, and approved submitted version.

## Funding

This study was supported by the Beijing Municipal Natural Science Foundation (Grant No. 9192004) and the National Natural Science Foundation of China (Grant Nos. 71974133 and 71573182).

## Conflict of Interest

The authors declare that the research was conducted in the absence of any commercial or financial relationships that could be construed as a potential conflict of interest.

## Publisher's Note

All claims expressed in this article are solely those of the authors and do not necessarily represent those of their affiliated organizations, or those of the publisher, the editors and the reviewers. Any product that may be evaluated in this article, or claim that may be made by its manufacturer, is not guaranteed or endorsed by the publisher.

## Abbreviations

SMD: severe mental disorders
WHO: world health organization.

## References

[1] Management of Physical Health Conditions in Adults With Severe Mental Disorders. World Health Organization (2018). p. 89. 30507109

[2] Coles BA. Intensive case management for severe mental illness. Issues Ment Health Nurs. (2018) 39:195–7. 10.1080/01612840.2017.1355184 28767005

[3] Baxter AJ Charlson FJ Somerville AJ Whiteford HA. Mental disorders as risk factors: assessing the evidence for the global burden of disease study. BMC Med. (2011) 9:134. 10.1186/1741-7015-9-134 22176705 PMC3305628

[4] Mental health. World Health Organization. E. coli. (2019). Available online at: https://www.who.int/news-room/facts-in-pictures/detail/mental-health (accessed October 5, 2021).

[5] Global Health Estimates: Leading Causes of DALYs. Mental health. World Health Organization. Available online at: https://www.who.int/data/gho/data/themes/mortality-and-global-health-estimates/global-health-estimates-leading-causes-of-dalys (accessed November 28, 2021).

[6] Alonso J Angermeyer MC Bernert S Bruffaerts R Brugha TS Bryson H. Disability and quality of life impact of mental disorders in Europe: results from the European study of the epidemiology of mental disorders (ESEMeD) project. Acta Psychiatr Scand Suppl. (2004) 420:38–46. 10.1111/j.1600-0047.2004.00329.x 15128386

[7] Mental Health-Burden. Available online at: https://www.who.int/health-topics/mental-health#tab=tab_2 (accessed November 21, 2021).

[8] Notice of the Health Commission on Printing and Distributing the Standards for the Management and Treatment of Severe Mental Disorders. Available online at: http://www.gov.cn/gongbao/content/2018/content_5338247.htm (accessed November 15, 2021).

[9] Zhu J Huang Q Lu W Chen Y Li B Xu Y. Do community free-medication service policy improve patient medication adherence? a cross-sectional study of patients with severe mental disorders in beijing community. Front Public Health. (2021) 9:714374. 10.3389/fpubh.2021.714374 34381755 PMC8351906

[10] Comprehensive Mental Health Action Plan 2013–2020. Available online at: https://apps.who.int/gb/ebwha/pdf_files/WHA66/A66_R8-en.pdf?ua=1 (accessed November 28, 2021).

[11] Good BJ Good MJ. Significance of the 686 Program for China and for global mental health. Shanghai Arch Psychiatry. (2012) 24:175–7. 10.3969/j.issn.1002-0829.2012.03.008 25324623 PMC4198850

[12] Drake RE Latimer E. Lessons learned in developing community mental health care in North America. World Psychiatry. (2012) 11:47–51. 10.1016/j.wpsyc.2012.01.007 22295009 PMC3266763

[13] Vanderplasschen W Rapp RC Pearce S Vandevelde S Broekaert E. Mental health, recovery, and the community. ScientificWorldJournal. (2013) 2013:926174. 10.1155/2013/926174 23576911 PMC3618936

[14] Chen FP Oh H. Staff views on member participation in a mental health clubhouse. Health Soc Care Community. (2019) 27:788–96. 10.1111/hsc.12697 30506799

[15] *Statistical Bulletin on the Development of Health Services in Beijing*. Beijing Municipal Health Commission Information Center (2021).

[16] Li AP Ma L Han J Zhang F Wang KL. A survey of self-care ability and nursing conditions of long-term hospitaIization of patients with mentaI disorders in different institutions of Chaoyang Region Beijing city. Chin Nursing Res. (2012) 26:2239. 10.3969/j.1009-6493.2012.24.015

[17] Chen HY Xie B Lin YQ Wang YY Xu HF. Cross-sectional studies on activity of daily living and social support for elderly patients with severe mental disorder in community. Shanghai J Prev Med. (2019) 31:154. 10.19428/j.cnki.sjpm.2019.19020

[18] Hartigan I. A comparative review of the Katz ADL and the barthel index in assessing the activities of daily living of older people. Int J Older People Nurs. (2007) 2:204–12. 10.1111/j.1748-3743.2007.00074.x 20925877

[19] Mayoral AP Ibarz E Gracia L Mateo J Herrera A. The use of barthel index for the assessment of the functional recovery after osteoporotic hip fracture: one year follow-up. PLoS ONE. (2019) 14:e0212000. 10.1371/journal.pone.0212000 30730973 PMC6366714

[20] Suwanmanee S Nanthamongkolchai S Munsawaengsub C Taechaboonsermsak P. Factors influencing the mental health of the elderly in Songkhla, Thailand. J Med Assoc Thai. (2012) 95:S8–15. 23130483

[21] Kiely KM Brady B Byles J. Gender, mental health and ageing. Maturitas. (2019) 129:76–84. 10.1016/j.maturitas.2019.09.004 31547918

[22] Taş S. Early period self-care ability and care requirements of schizophrenia patients after discharge. J Psychiatric Nurs. (2017) 9:11–22. 10.14744/phd.2017.64935

[23] Holmqvist KL Holmefur M. The ADL taxonomy for persons with mental disorders - adaptation and evaluation. Scand J Occup Ther. (2019) 26:524–34. 10.1080/11038128.2018.1469667 29720019

[24] Mlinac ME Feng MC. Assessment of activities of daily living, self-care, and independence. Arch Clin Neuropsychol. (2016) 31:506–16. 10.1093/arclin/acw049 27475282

[25] Berlim MT Mattevi BS Duarte AP Thomé FS Barros EJ Fleck MP. Quality of life and depressive symptoms in patients with major depression and end-stage renal disease: a matched-pair study. J Psychosom Res. (2006) 61:731–4. 10.1016/j.jpsychores.2006.04.011 17084154

[26] Kuehner C Buerger C. Determinants of subjective quality of life in depressed patients: the role of self-esteem, response styles, and social support. J Affect Disord. (2005) 86:205–13. 10.1016/j.jad.2005.01.014 15935240

[27] Peltzer K Phaswana-Mafuya N. Depression and associated factors in older adults in South Africa. Glob Health Action. (2013) 6:1–9. 10.3402/gha.v6i0.18871 23336621 PMC3549465

[28] Skevington SM McCrate FM. Expecting a good quality of life in health: assessing people with diverse diseases and conditions using the WHOQOL-BREF. Health Expect. (2012) 15:49–62. 10.1111/j.1369-7625.2010.00650.x 21281412 PMC5060606

[29] Fleury MJ Grenier G Bamvita JM Tremblay J Schmitz N Caron J. Predictors of quality of life in a longitudinal study of users with severe mental disorders. Health Qual Life Outcomes. (2013) 11:92. 10.1186/1477-7525-11-92 23758682 PMC3681595

[30] Shumye S Amare T Derajew H Endris M Molla W Mengistu N. Perceived quality of life and associated factors among patients with severe mental illness in Ethiopia: a cross-sectional study. BMC Psychol. (2021) 9:152. 10.1186/s40359-021-00664-w 34602067 PMC8489038

[31] Clarke DM Kissane DW. Demoralization: its phenomenology and importance. Aust N Z J Psychiatry. (2002) 36:733–42. 10.1046/j.1440-1614.2002.01086.x 12406115

[32] Verhaeghe N De Maeseneer J Maes L Van Heeringen C Annemans L. Health promotion in mental health care: perceptions from patients and mental health nurses. J Clin Nurs. (2013) 22:1569–78. 10.1111/jocn.12076 23294398

[33] Yanos PT Roe D Lysaker PH. The impact of illness identity on recovery from severe mental illness. Am J Psychiatr Rehabil. (2010) 13:73–93. 10.1080/15487761003756860 20802840 PMC2927828

[34] Silva Junior JS Fischer FM. Disability due to mental illness: social security benefits in Brazil 2008-2011. Rev Saude Publica. (2014) 48:186–90. 10.1590/S0034-8910.2014048004802 24789650 PMC4206132

[35] Lan Y. The Study for Inpatient Expense of Severe Mental Diseases Patients and Its Influencing Factors in Qufu. Shandong University (2015).

[36] Caron J Mercier C Diaz P Martin A. Socio-demographic and clinical predictors of quality of life in patients with schizophrenia or schizo-affective disorder. Psychiatry Res. (2005) 137:203–13. 10.1016/j.psychres.2005.07.002 16298428

[37] Parabiaghi A Bonetto C Ruggeri M Lasalvia A Leese M. Severe and persistent mental illness: a useful definition for prioritizing community-based mental health service interventions. Soc Psychiatry Psychiatr Epidemiol. (2006) 41:457–63. 10.1007/s00127-006-0048-0 16565917

[38] Essau CA de la Torre-Luque A. Comorbidity profile of mental disorders among adolescents: a latent class analysis. Psychiatry Res. (2019) 278:228–34. 10.1016/j.psychres.2019.06.007 31226549

[39] Sartorius N. Comorbidity of mental and physical disorders: a key problem for medicine in the 21st century. Acta Psychiatr Scand. (2018) 137:369–70. 10.1111/acps.12888 29637546

[40] van Buitenen N van den Berg CJW Meijers J Harte JM. The prevalence of mental disorders and patterns of comorbidity within a large sample of mentally ill prisoners: a network analysis. Eur Psychiatry. (2020) 63:e63. 10.1192/j.eurpsy.2020.63 32522312 PMC7355171

[41] AGHA MT Tavafian SS Zare S. Health related quality of life in elderly people living in Bandar Abbas, Iran: a population-based study. Acta Med Iran. (2010) 48:185–91. 21137656

[42] Ansari E Mishra S Tripathi A Kar SK Dalal PK. Cross-sectional study of internalised stigma and medication adherence in patients with obsessive compulsive disorder. Gen Psychiatr. (2020) 33:e100180. 10.1136/gpsych-2019-100180 32215363 PMC7066600

[43] García-Pérez L Linertová R Serrano-Pérez P Trujillo-Martín M Rodríguez-Rodríguez L Valcárcel-Nazco C. Interventions to improve medication adherence in mental health: the update of a systematic review of cost-effectiveness. Int J Psychiatry Clin Pract. (2020) 24:416–27. 10.1080/13651501.2020.1782434 32609024

[44] Li H Lu GQ Shen QR. Study on the relationship between stigma and medication compliance of bipolar disorder in convalescence. Contemp Med. (2021) 27:181–2.

[45] Liu HJ. Analysis of effect of continuous nursing on drug compliance in schizophrenic patients. Guide Chin Med. (2021) 19:169–70.

[46] Schulze LN Stentzel U Leipert J Schulte J Langosch J Freyberger HJ. Improving medication adherence with telemedicine for adults with severe mental illness. Psychiatr Serv. (2019) 70:225–8. 10.1176/appi.ps.201800286 30651059

[47] Sun KS Lam TP Lam KF Lo TL. Barriers and facilitators for psychiatrists in managing mental health patients in Hong Kong-Impact of Chinese culture and health system. Asia Pac Psychiatry. (2018) 10:1–7. 10.1111/appy.12279 28371455

[48] Munikanan T Midin M Daud TIM Rahim RA Bakar AKA Jaafar NRN. Association of social support and quality of life among people with schizophrenia receiving community psychiatric service: a cross-sectional study. Compr Psychiatry. (2017) 75:94–102. 10.1016/j.comppsych.2017.02.009 28342379

[49] Olalemi OE Muyibi SA Ladipo MM. Perceived family support and medication adherence amongst hypertensive outpatients in a tertiary hospital, Ibadan, Nigeria. West Afr J Med. (2020) 37:481–9. 33058123

[50] Aftab A Joshi Y Sewell D. Conceptualizations of mental disorder at a US academic medical center. J Nerv Ment Dis. (2020) 208:848–56. 10.1097/NMD.0000000000001227 32947448

[51] Auerbach RP Mortier P Bruffaerts R Alonso J Benjet C Cuijpers P. WHO world mental health surveys international college student project: prevalence and distribution of mental disorders. J Abnorm Psychol. (2018) 127:623–38. 10.1037/abn0000362 30211576 PMC6193834

[52] Humpston CS Bebbington P Marwaha S. Bipolar disorder: prevalence, help-seeking and use of mental health care in England. Findings from the 2014 Adult Psychiatric Morbidity Survey. J Affect Disord. (2021) 282:426–33. 10.1016/j.jad.2020.12.151 33422818

[53] Chauhan SK Dhar M. Prevalence and predictors of mental health disorder among the adolescent living in the slums of lucknow, India: a cross-sectional study. Community Ment Health J. (2020) 56:383–92. 10.1007/s10597-019-00452-2 31531783

