# Supplementary material for: Self-Care Ability of Patients With Severe Mental Disorders: Based on Community Patients Investigation in Beijing, China
Source: Front Public Health. 2022 Jun 1;10:847098. doi: 10.3389/fpubh.2022.847098 (PMC9198226; doi:10.3389/fpubh.2022.847098)
Supplement: Supplementary file 1 [file Table_1.DOCX]

This is a Multimedia Appendix to a full manuscript published in the J Med Internet Res. For full copyright and citation information see http://dx.doi.org/10.2196/jmir.xxxx

**问卷编号：**_______________

**监护人编号：_______________登记的监护人没来参加调查的原因：**

北京市严重精神障碍患者自理能力现状评估调查问卷

您好，我们是北京大学的师生，受北京市卫生健康委员会的委托，目前正在进行一项关于北京市严重精神障碍患者自理能力现状情况的调研，主要目的是了解您所照护的精神障碍患者自理能力状况，以及您和患者关于疾病康复的需求及问题，以便向政府有关部门提供建议，更好地完善相关政策。您的回答对于我们非常重要，答案也没有对错之分，请您根据自己的实际情况和感受填写。本次调查的信息资料仅用于研究，并将严格进行保密，所以请您不要有任何顾虑。如果您有任何问题，请及时与我们项目组沟通，联系方式是13691070267。衷心感谢您的支持与合作！祝您身体健康，生活愉快！

**注意事项：**（1）本问卷共9页，**正反两面**印刷，请大家不要漏答；

（2）请用**黑色/蓝色**的圆珠笔或中性笔填写；

（3）除标记为**多选**的题外，其余均为**单选题**，请在选项序号上**画圈**。

**以下内容由调查员填写：**

**调查员**：_______________

**区:**_______________ **街道**_______________ **社区**_______________

**调查日期：** 年 月 日

**调查开始时间：**_____时______分 **调查结束时间：**_______时_ _分

**是否合格：**现场 ______________复核____________录入____________

**以下内容由调查员填写：**

**调查员**：_______________

**区:**_______________ **街道**_______________ **社区**_______________

**调查日期：** 年 月 日

**调查开始时间：**_____时______分 **调查结束时间：**_______时_ _分

**是否合格：**现场 ______________复核____________录入____________

# A 监护人的基本信息

A1您的性别是:

1.男 2.女

A2您的出生年月是__________年__________月

A3您的户籍性质是:

1.北京城镇户口 2.北京农村户口 3.外地城镇户口 4.外地农村户口

A4您的受教育程度为:

1.未正式上过学（**包括上私塾或夜校、培训班**） 2.小学 3.初中

4.高中/中专/职高 5.大专 6.本科及以上

A5您目前身体健康状况如何:

1很好 2好 3.一般 4.不好 5.很不好

A6您目前的精神状况如何？

1很好 2好 3.一般 4.不好 5.很不好

# B 患者的基本信息

B1患者的性别是:

1.男 2.女

B2患者的出生年月是__________年__________月

B3患者的户籍性质是:

1.北京城镇户口 2.北京农村户口 3.外地城镇户口 4.外地农村户口

B4患者的受教育程度为:

1.未正式上过学（**包括上私塾或夜校、培训班**） 2.小学 3.初中 4.高中/中专/职高5.大专 6.本科及以上

B5患者是哪一年开始患病的？__________年。**（以诊断时间为准）**

B6患者所患的疾病是：

1精神分裂症 2. 双相情感障碍 3. 精神发育迟滞伴发精神障碍 4. 妄想性障碍(偏执性精神病)，分裂情感性障碍，癫痫所致精神障碍 5. 非六类国家强报

B7.请您回忆下，申请补贴之前，患者的整体状况如何：

|  | 很低 | 较低 | 一般 | 较高 | 很高 |
| --- | --- | --- | --- | --- | --- |
| 服药依从性（**配合服药**） | 1 | 2 | 3 | 4 | 5 |
| 精神稳定性 | 1 | 2 | 3 | 4 | 5 |
| 康复活动参与程度 | 1 | 2 | 3 | 4 | 5 |

B8患者目前是否有其他疾病？

1.是：

2.否

B9患者目前的工作/劳动状态是：

1.正常退休未再就业 2.病退/内退未再就业 3.全职 4.兼职 5.挂靠

6.失业（**以前工作过，现在没有工作**） 7.无业（**从未工作过**） 8.其他______

B10.1患者每周工作__________小时。

B10.2患者工作/退休金的年收入为__________元。

B11患者的家庭年收入为__________元。（**不包括福利性收入**）

B12患者的家庭的主要收入来源为：（单选题）

1.工资性收入、劳动报酬 2.退休金 3.社会保障金及救济金 4.财产性收入 5.亲属给钱 6.其他__________

# C 监护人的监护与照顾信息

C1患者是您的什么亲属:

1配偶 2 父母 3子女 4孙子女 5兄弟姐妹 6亲戚 7朋友 8.其他

C2您从哪一年开始照顾患者？ 年.

C3目前，您除了照顾患者，还要照顾下列哪些人？**（可多选）**

□未照顾别人 □配偶 □父母 □子女 □孙子女 □兄弟姐妹 □亲戚 □朋友 □其他

C4.目前家中谁跟您一起照顾患者？**（可多选）**

□患者的父母 □患者的配偶 □患者的子女 □患者的兄妹 □其他亲戚

□朋友 □保姆 □护工 □只有您在照顾 □其他

C5患者的日常生活能力，监护人的监护内容，以及提供帮助、进行监护的时间分配**（请注意时间单位）**

| 患者的自理能力评价 | | | | 监护人提供帮助花费的时间 |
| --- | --- | --- | --- | --- |
| 内容 | 患者完全不能做 | 患者需要他人帮助才能做 | 患者完全自己能做 |  |
| 吃饭 | 1 | 2 | 3 | **日常基本活动**  小时/天 |
| 穿衣服 | 1 | 2 | 3 |  |
| 梳头、刷牙、剪指甲 | 1 | 2 | 3 |  |
| 洗澡 | 1 | 2 | 3 |  |
| 上厕所 | 1 | 2 | 3 |  |
| 上下床 | 1 | 2 | 3 |  |
| 室内走动 | 1 | 2 | 3 |  |
| 上下楼梯 | 1 | 2 | 3 |  |
| 自己吃药 | 1 | 2 | 3 |  |
| 扫地 | 1 | 2 | 3 | **家务活动**  小时/天 |
| 做饭 | 1 | 2 | 3 |  |
| 洗衣 | 1 | 2 | 3 |  |
| 日常购物 | 1 | 2 | 3 | **社会活动**  小时/月 |
| 打电话 | 1 | 2 | 3 |  |
| 管理财务 | 1 | 2 | 3 |  |
| 使用公交交通 | 1 | 2 | 3 |  |
| 到家附近的地方 | 1 | 2 | 3 |  |
| 参与康复活动 | 1 | 2 | 3 |  |

**本次问卷调查到此结束，感谢您的参与！**
